# Comparative Whole-Genomic Analysis of an Ancient L2 Lineage *Mycobacterium tuberculosis* Reveals a Novel Phylogenetic Clade and Common Genetic Determinants of Hypervirulent Strains

**DOI:** 10.3389/fcimb.2017.00539

**Published:** 2018-01-12

**Authors:** Rahim Rajwani, Wing Cheong Yam, Ying Zhang, Yu Kang, Barry Kin Chung Wong, Kenneth Siu Sing Leung, Kingsley King Gee Tam, Ketema Tafess Tulu, Li Zhu, Gilman Kit Hang Siu

**Affiliations:** ^1^Department of Health Technology and Informatics, The Hong Kong Polytechnic University, Hong Kong, Hong Kong; ^2^Department of Microbiology, Li Ka Shing Faculty of Medicine, The University of Hong Kong, Hong Kong, Hong Kong; ^3^Department of Molecular Microbiology and Immunology, Bloomberg School of Public Health, Johns Hopkins University, Baltimore, MD, United States; ^4^CAS Key Laboratory of Genome Sciences and Information, Beijing Institute of Genomics, Chinese Academy of Sciences, Beijing, China; ^5^Department of Pathology, United Christian Hospital, Hong Kong, Hong Kong

**Keywords:** *Mycobacterium tuberculosis*, hypervirulent, macrophage, Pacbio, lineage2, comparative genomics, phylogenetic, virulence

## Abstract

**Background:** Development of improved therapeutics against tuberculosis (TB) is hindered by an inadequate understanding of the relationship between disease severity and genetic diversity of its causative agent, *Mycobacterium tuberculosis*. We previously isolated a hypervirulent *M. tuberculosis* strain H112 from an HIV-negative patient with an aggressive disease progression from pulmonary TB to tuberculous meningitis—the most severe manifestation of tuberculosis. Human macrophage challenge experiment demonstrated that the strain H112 exhibited significantly better intracellular survivability and induced lower level of TNF-α than the reference virulent strain *H37Rv* and other 123 clinical isolates.

**Aim:** The present study aimed to identify the potential genetic determinants of mycobacterial virulence that were common to strain H112 and hypervirulent *M. tuberculosis* strains of the same phylogenetic clade isolated in other global regions.

**Methods:** A low-virulent *M. tuberculosis* strain H54 which belonged to the same phylogenetic lineage (L2) as strain H112 was selected from a collection of 115 clinical isolates. Both H112 and H54 were whole-genome-sequenced using PacBio sequencing technology. A comparative genomics approach was adopted to identify mutations present in strain H112 but absent in strain H54. Subsequently, an extensive phylogenetic analysis was conducted by including all publically available *M. tuberculosis* genomes. Single-nucleotide-polymorphisms (SNPs) and structural variations (SVs) common to hypervirulent strains in the global collection of genomes were considered as potential genetic determinants of hypervirulence.

**Results:**Sequencing data revealed that both H112 and H54 were identified as members of the same sub-lineage L2.2.1. After excluding the lineage-related mutations shared between H112 and H54, we analyzed the phylogenetic relatedness of H112 with global collection of *M. tuberculosis* genomes (*n* = 4,338), and identified a novel phylogenetic clade in which four hypervirulent strains isolated from geographically diverse regions were clustered together. All hypervirulent strains in the clade shared 12 SNPs and 5 SVs with H112, including those affecting key virulence-associated loci, notably, a deleterious SNP (*rv0178* p. D150E) within *mce1* operon and an intergenic deletion (854259_ 854261delCC) in close-proximity to *phoP*.

**Conclusion:** The present study identified common genetic factors in a novel phylogenetic clade of hypervirulent *M. tuberculosis*. The causative role of these mutations in mycobacterial virulence should be validated in future study.

## Introduction

Despite the continuous effort to combat tuberculosis (TB) in the past few decades, it remains to be a major health problem globally. In particular, the challenge from TB is worsened by the rapid emergence of multidrug resistant (MDR) and even extensively drug-resistant (XDR) TB cases. Development of new drugs and vaccines has proved difficult owing to our poor understanding of the pathogenesis of the causative agent, *Mycobacterium tuberculosis*.

*M. tuberculosis* is an intracellular pathogen that is able to modulate the host immune response and persist inside the macrophage, leading to a latent infection with limited replicative potential (Smith, 2003). However, previous studies demonstrated that some clinical strains have established virulence mechanisms that disrupt the delicate balance between replication and survival of its host. They multiply rapidly inside the host macrophage, followed by escaping from immune barriers in the lungs and spreading to other organs, causing more severe forms of the disease, such as tuberculous meningitis, even in immunocompetent individuals (Caminero et al., 2001; Alonso et al., 2011; Ribeiro et al., 2014).

In our previous study, virulence of 125 *M. tuberculosis* clinical strains was evaluated by measuring intracellular survival in peripheral-blood-monocyte-derived-macrophages (PBMDMs), as well as quantifying the release of pro-inflammatory cytokines (TNFα, IL-10, and IL-12) upon infection (Wong et al., 2007). Interestingly, one of the strains (H112) was shown to grow more rapidly and induce lower level of TNF-α than a large number of other strains, consistently in different batches of PBMDMs collected from multiple healthy donors (Wong et al., 2007). Moreover, clinical record revealed that H112 was isolated from the cerebrospinal fluid of an HIV-negative patient who had aggressive disease progression from pulmonary TB to tuberculous meningitis within 2 months. We hypothesized that the genome *M. tuberculosis H112* encodes an altered set of virulence-factors that enables it to survive within the hostile macrophage environment more efficiently than other strains.

The current study aims to investigate the genomic uniqueness of hypervirulent strain H112 by a two-step comparative genomic approach. The first step focuses on masking lineage-related genetic polymorphisms by a comparison with a genetically-related less-virulent strain, H54. The second step concentrates on identifying a subset of H112-specific mutations that are commonly found in hypervirulent strains reported elsewhere.

## Materials and methods

### Ethical approval and biological safety

This study has been approved by the Institutional Review Board of The Hong Kong Polytechnic University (Ref. number: RSA15096). All experiment involving viable *M. tuberculosis* culture were handled in biosafety level 3 (BSL-3) laboratory with an approved protocol (HSE(HKU)_20160104).

### *M. tuberculosis* strains

In our previous study, 125 *M. tuberculosis* clinical isolates collected in Hong Kong and Shanghai, China, between 2002 and 2004, were determined for the intra-macrophage survivabilities in PBMDMs and induction of pro-inflammatory cytokines by macrophages (Wong et al., 2007). A hypervirulent strain, H112, which demonstrated enhanced intracellular growth relative to 123 other strains was included in the present study. The strain was isolated from cerebrospinal fluid of a tuberculous meningitis patient who was a 51-year old male with no known comorbidity and was HIV-negative. The patient was first diagnosed as pulmonary TB in April 2004, but then failed to comply with anti-TB treatment course. The disease rapidly progressed into tuberculous meningitis 2 months later. The patient died in July 2004. *In vitro* drug susceptibility testing revealed that the strains were pan-susceptible to all first-line anti-TB drugs. Subsequently, another clinical isolate, H54, which belonged to the same phylogenetic lineage as the hypervirulent strain, was obtained from respiratory specimen of a newly diagnosed pulmonary TB patient. The patient was a 67-year old, HIV negative male admitted to the same clinical center in Hong Kong in October 2004. He was successfully cured upon the completion of a standard course of first-line anti-TB treatment. Strain H54 was used as comparator of the hypervirulent strain H112 in whole-genome sequence analysis in this study. *M. tuberculosis* reference strains H37Rv (ATCC 27294) and H37Ra (ATCC 25177) were purchased from American type tissue culture collection (ATCC) as virulent and avirulent control strains respectively for THP-1 cell challenge experiment.

### MIRU-VNTR typing

Frozen bacterial stocks were subcultured on Middlebrook 7H10 Agar to obtain isolated colonies. DNA was extracted from isolated colonies using Cobas Amplicor Respiratory kit (Roche Diagnostics, Germany) as previously described (Siu et al., 2011). Each of the 12 loci MIRU were amplified individually and resolved on 2.5% agarose gel electrophoresis to infer number of repeats at each locus based on amplicon size as described previously (Cowan et al., 2002). Phylogenetic analysis of the 12 loci MIRU was conducted using MIRU-VNTR plus (Weniger et al., 2010).

### Spoligotyping

Prior to WGS, spoligotyping was performed according to standardized laboratory procedure described previously (Kamerbeek et al., 1997). The resulting spoligotypes were used to assign *M. tuberculosis* lineages to strains in TB-lineage (Shabbeer et al., 2012).

### Infection of THP-1 cells with *Mycobacterium tuberculosis* and measurement of growth index

The THP-1 monocytic cell line (ATCC TIB-202) was cultured at density 3 × 10^8^ cells per well into 24-well plate containing RMI1640 medium (GIBCO, USA) supplemented with 5% (v/v) fetal bovine serum (GIBCO, USA) at 37°C with 5% CO_2._ Viability of cells was assessed by staining with 0.4% (w/v) Trypan blue and differentiation was induced overnight by treatment with 20 nM phorbol 12-myristate 13-acetate (Sigma Aldrich, USA). The THP-1 cells were rested for 24 h by incubating in RMI1640 without PMA before infecting with mycobacterial strains. Differentiated THP-1 cells were then infected at a multiplicity of infection (MOI) of 1:1 overnight with *M. tuberculosis* strains pre-passaged through 25-guage needle. Extracellular bacteria were removed by washing three times with RMI1640 medium. The cells were lysed at day 0, 1, 3, and 6 using 0.1% (v/v) sodium dodecyl sulfate (SDS) and 10-fold diluted suspensions of intracellular bacteria were plated on Middlebrook 7H10 agar (DIFCO, USA) supplemented with 10% (v/v) oleic acid-albumin-dextrose-catalase (Becton Dickinson, USA). Colony forming units per ml were enumerated after 4 weeks. The experiment was performed in triplicate and mean CFU per ml was calculated. Growth index was determined as mean CFU per ml at day x divided by mean CFU per ml at day 0.

### DNA extraction

Genomic DNA for Pac-Bio sequencing was isolated as described previously (Benjak et al., 2015). Briefly, 10 ml of late-log phase culture of *M. tuberculosis* strain was prepared in 7H9 medium which was then pelleted and frozen at −80°C overnight. Pellet was resuspended into SET buffer (25% w/v sucrose, 50 mM EDTA, 50 mM Tris-HCL, pH:8.0) with the addition of lysozyme and incubated at 37°C overnight. This was followed by treatment with proteinase K and RNAase. DNA was purified once through Phenol-Chloroform-isoamyl alcohol (25:24:1) and secondly through chloroform-isoamyl alcohol (24:1) layer. Quality and integrity of DNA was checked through Qubit HS assay (Thermo Scientific, USA) and 0.8% agarose gel electrophoresis respectively.

### Single molecule real-time (SMRT) sequencing, *de novo* assembly, and annotation

A total of 15 μg of genomic DNA (gDNA) from each *M. tuberculosis* strain was used to prepare 20-Kb libraries which were sequenced by P6-C4 chemistry using one SMRT cell per library (Pacific biosciences, USA). The library was loaded using MagBead One Cell per Well (OCPW version 1) protocol to capture data in 240 min movie time. *De novo* assembly from the resulting continuous long reads (CLR) was performed using Hierarchical Genome Assembly Process (HGAP.2) algorithm from SMRT portal (version 2.3.0) (Chin et al., 2013).

### Phylogenetic analysis and SNP detection

Previously published assembled genomes (*n* = 22) from all eight *M. tuberculosis complex* (MTBC) lineages were downloaded from NCBI GenBank (Table S1). A multiple core-genome alignment was generated using Parsnp utility in Harvest Suite (Treangen et al., 2014). The alignment was then used to construct maximum-likelihood (ML) tree using generalized time reversible (GTR), proportion of invariable sites 0.0 and number of substitutions per categories as 4 in MEGA7 (Kumar et al., 2016). A multi-sample variant-call-file (VCF) was generated from core-genome alignment and SNPs present in H112 but absent in H54 were extracted using BCFtools (Li et al., 2009).

For detailed phylogenetic analysis, a total of 4,335 assembled genomes of *M. tuberculosis* in NCBI-Genbank (accessed on 8/12/2016) and un-assembled genome data-sets from previous studies (Zhang et al., 2013; Luo et al., 2015; Merker et al., 2015) were searched for lineage 2 strains harboring at least one of the H112-specific SNPs (i.e., SNPs present in H112 but absent in the comparator strain H54) (Table S2).

Subsequently, raw reads were aligned to the reference genome (Genbank accession NC_000962.3) using Bowtie2 and SNPs with depth greater than 5, allele frequency exactly 1, mapping quality greater than 20, and Phred-scaled variant quality in excess of 20 were called using SAMtools (Li et al., 2009). A list of all informative SNP positions was compiled and corresponding base calls for all samples were retrieved. SNP loci with missed call in any of the samples were discarded to obtain list of highly credible phylogenetically informative SNPs. Concatenated SNPs was used to construct ML tree using GTR model in MEGA7 as described above (Kumar et al., 2016).

As a quality-filtering step, nearly identical genomes (pair-wise SNP differences less than eight) or unusually long-branched on the phylogenetic tree (indicating sequencing-errors) were excluded. The final data-set comprised of 33 strains from 13 countries. Statistical support for clades was determined using bootstrap analysis with 100 pseudo-replicates. Trees were visualized in FigTree v1.4.3.

Furthermore, MSA was used to conduct principal component analysis (PCA) and compute pair-wise SNP distance within and between groups in JalView and MEGA7 respectively.

### Predicting impact of non-synonymous SNPs

Deleterious impact of mutations on protein function was predicted using sorting intolerant from tolerant (SIFT 4G) (Kumar et al., 2009). Predictions with a score <0.05 were considered significant.

### Detection of structural variations

Structural variations (SVs, ranging 1–10,000 bp) present in H112 but absent in H54 were identified using Assemblytics (Nattestad and Schatz, 2016). Briefly, genome assemblies were aligned against the reference genome *M. tuberculosis H37Rv* (NC_000962.3) using NUCmer with –minmatch 100 and –mincluster 500. The alignment file was used to call structural variations within and between alignments with at least 100 bp unique anchor sequence in Assemblytics (Nattestad and Schatz, 2016). SVs that do not overlap between H112 and H54 were identified using BEDtools (Quinlan and Hall, 2010). Presence of identified SVs in short-read sequencing data (used in detailed phylogenetic analysis) was verified by manual inspection of binary alignment map (BAM) files.

In addition, SVs mediated by *IS6110* insertion were identified using IS-seeker (Adams et al., 2016). BLASTN was used to map complete or partial sequence of *IS6110* element (Genbank accession X17348). Sequences flanking the mapped regions were retrieved and aligned to the reference genome (NC_000962.3) for annotation.

### Genomic data availability

The genome sequencing data for strains sequenced in this study are deposited under NCBI Bio-Project accession number: PRJNA369711 (H112 accession CP019613; H54 accession CP019610).

## Results

### Selection of control strain to mask lineage-specific variability in comparative genomic analysis

**Figure 1:**
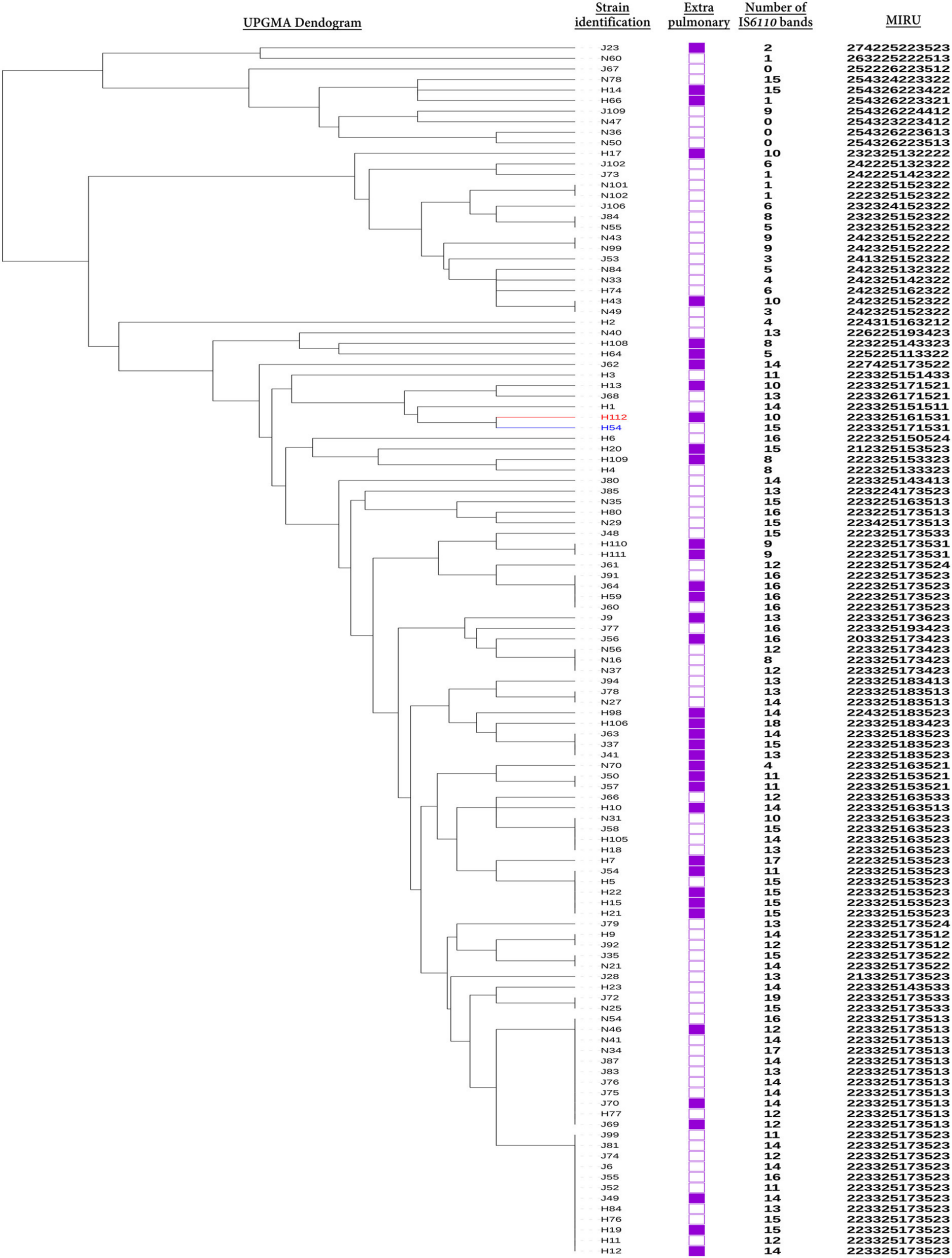
Selection of control strain. The relationship between Hypervirulent strain H112 (Red), control strain H54 (blue), and other 113 strains is indicated via dendogram. The dendogram was constructed using unweighted pair group method with arithmetic mean (UPGMA) algorithm based on MIRU. Additional information related to source of all strains that is extra-pulmonary (filled squares) or pulmonary TB (empty squares), number of *IS6110*-RFLP bands and MIRU patterns are also illustrated.

All available (*n* = 115/125;92%) isolates from previous study were cultivated from the frozen stocks and were subjected to DNA-fingerprinted based on polymorphisms within 12 MIRU loci. UPGMA tree-based analysis identified that hypervirulent strain H112 was genetically closest to H54 among other 113 strains. Genetic relationship between H112 and H54 was further confirmed by spoligotyping. Spoligotypes for both strains were found to be identical (000000000003771) and representative of lineage 2 (L2) (Figure 1). Together, both MIRU and spoligotype patterns indicated that H54 is genetically related to hypervirulent strain H112. Therefore, H54 was selected as a control strain to mask lineage-related genetic variation in subsequent comparative genomic analysis.

### Reassessment of intracellular growth in macrophage

**Figure 2:**
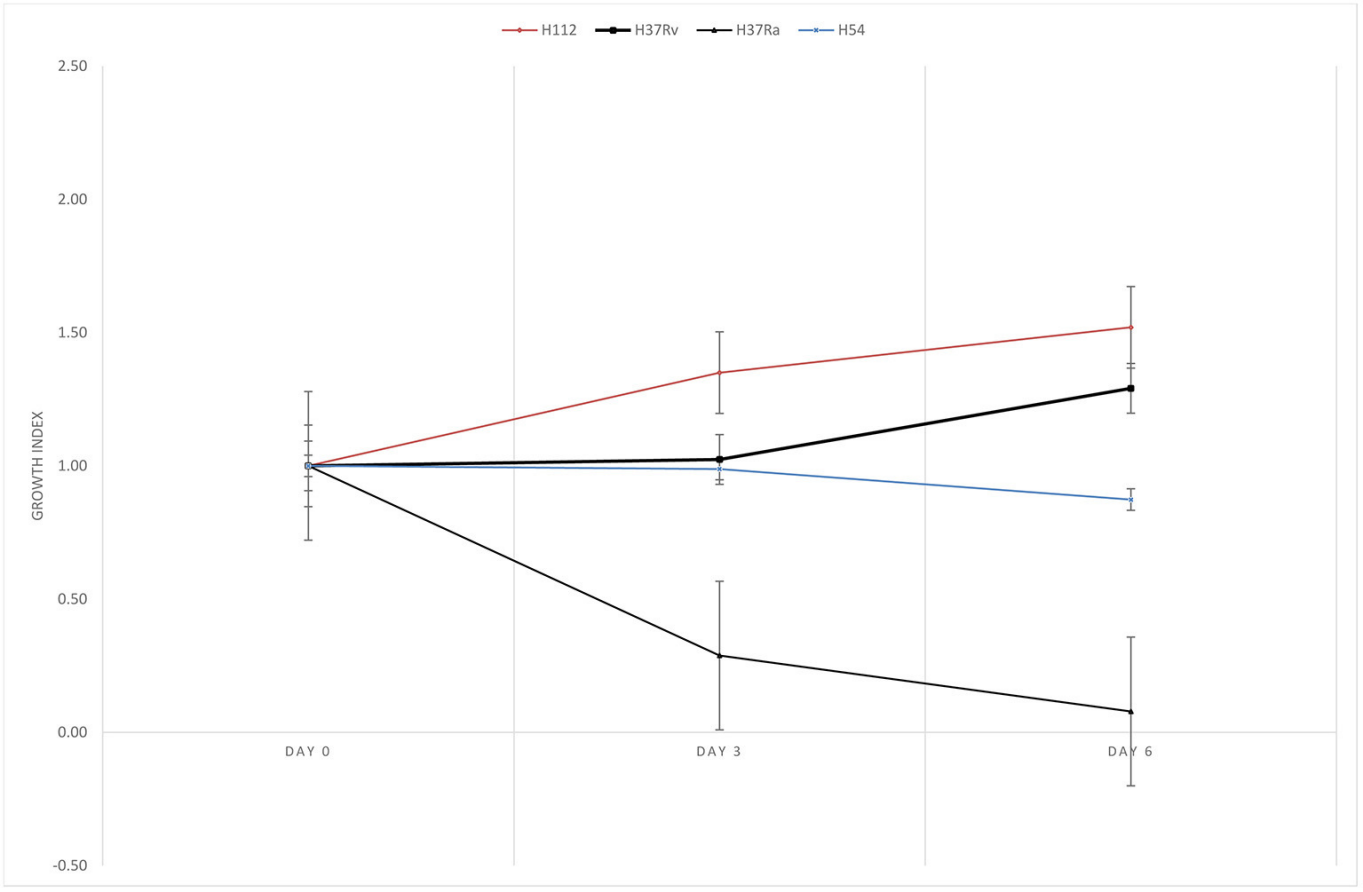
Reassessment of enhanced intracellular growth in THP-1 macrophage. Growth indexes showing multiplication of *M. tuberculosis* strains inside THP-1 macrophage. Higher growth index of hypervirulent strain H112 (red) relative to reference virulent strain H37Rv (black with squares), avirulent strain H37Ra (black with triangles), and control strains H54 (blue) were clearly demonstrated. Growth index was determined as mean CFU per ml at day 0, 3, 6 divided by mean CFU per ml at day 0, respectively. The lines represent mean of triplicate experiments.

H112 was demonstrated to have better survivability in PBMDMs in previous study (Wong et al., 2007). In present study, the ability of intracellular growth of H112 and H54 was reassessed and compared, as well as the reference strains in THP-1. The growth indices of *M. tuberculosis* strains inside THP-1 are shown in Figure 2. It was observed that shortly after infection (day 0–3), the bacterial counts of H112 increased, which indicated excellent adaptation to the harsh intracellular environment in human macrophage. On the contrary, the bacterial counts of H37Rv, H37Ra, and H54 remained constant or declined indicating reduced fitness of these strains accompanied by an early killing phase that macrophages attempted to eradicate the infection. This was followed by a second period (day 3–6) during which the cell number of H112 elevated further whereas the survival of H54 slightly declined. At day six after infection, both H112 and H37Rv showed 30–50% increase in cell numbers, confirming to their virulent phenotype, whereas H54 and H37Ra reduced in cell numbers by 12 and 92% respectively (Figure 2). Overall, the intracellular replication potential of H112 was found to be higher than H37Rv and clinical isolate of same major lineage, establishing itself as the hypervirulent strain.

### Whole-genome sequencing and *de novo* assembly

The sequencing run yielded high-quality (quality score > 0.8) reads with sub-reads N-50 value greater than 10 Kb. Interestingly, fully closed circular genome assembled into single contig with an average sequencing depth of ~150X was obtained for both *M. tuberculosis* strains. Size of H112 and H54 genomes was nearly same (~4.4 megabases) (Table 1).

**Table 1:** Results of whole-genome sequencing and assembly.

**Properties**	**Strain**	
	**H112**	**H54**
Number of subreads^a^	69322	77709
Mean subread length (bp)	7201	9585
Subreads N50 (bp)	10907	14058
Number of contigs	1	1
Genome length (Mb)	4.40	4.41
GC content (%)	65.62	65.61
Average read depth^b^	~113X	~168X

a *Number of filtered subreads passing minimum polymerase read quality (>0.8) used for de novo assembly*.

b Average read depth = Number of reads ^*^ read length/ genome size. (Lander/Waterman equation.)

### Phylogenetic placement using WGS

**Figure 3:**
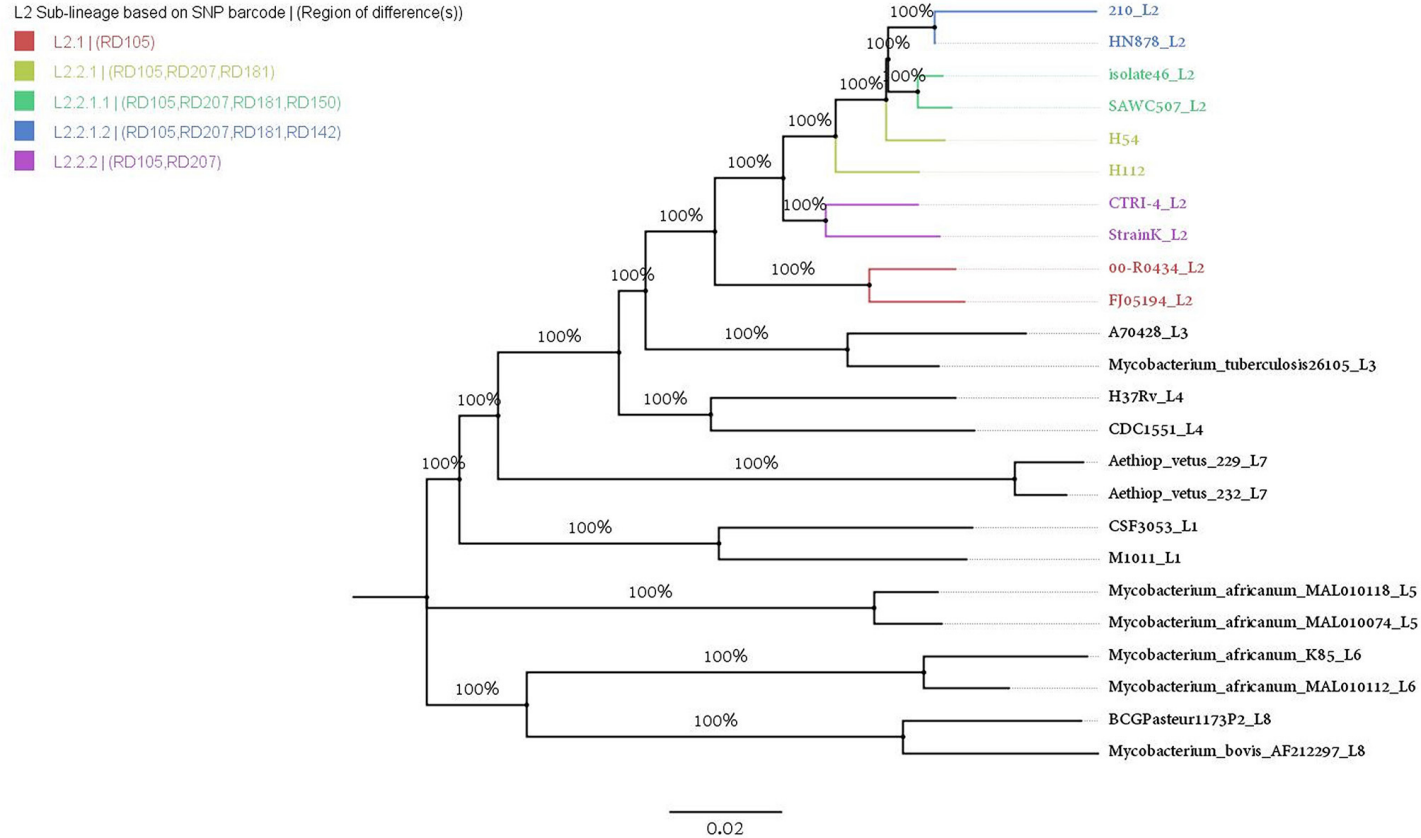
Phylogenetic placement of H112 and H54. Assignment of phylogenetic lineage to H112 and H54 is illustrated using ML-tree with representative strains from all eight major lineages (L1–L8) of *M. tuberculosis* complex (MTBC) and L2 sub-lineages. Branch labels indicate bootstrap values in percentage from 100 pseudo-replicates.

In order to contextualize phylogenetic placement of H112 and H54 in the global phylogeny of *Mycobacterium tuberculosis complex* (MTBC), phylogenetic tree was reconstructed with the inclusion of representative genomes from all L2 sub-lineages (*n* = 8) and other MTBC major lineages (*n* = 14) (Figure 3). It was observed that H112 and H54 were represented by adjacent branches on the tree. Moreover, these branches were enclosed within a clade representative of L2 (Figure 3). According to SNP-based classification scheme for *M. tuberculosis* (Coll et al., 2014), H112 and H54 were identified as members of the same sub-lineage L2.2.1, which was also known as ancient L2. The sub-lineage assignment was also corroborated by absence of region-of-difference (RD)105, RD207, and RD181 in both strains H112 and H54. Overall, consistent with results obtained using conventional genotyping, H112 and H54 were found to be genetically-related at sub-lineage level.

### Comparative genomics between hypervriulent strain H112 and control strain H54 within phylogenetic framework

#### Single nucleotide polymorphisms

There were 1,238 SNPs associated with hypervirulent strain H112. Interestingly, a vast majority (1,099/1,238; 88.7%) of H112-associated SNPs were common with H54 (Figure S1). SNPs shared between H112 and H54 constitute genetic variation related to lineage. For instance, SNPs accumulated as a member of L2.2.1 (85/1,099; 7.7%), L2.2 (111/1,099; 10.1%), L2 (112/1,099; 10.1%), and so on. There were 139 SNPs (139/1,238; 11.2%) present only in H112 (Table S3). Out of 139, 11 (11/139; 7.9%) were intergenic, 85 (85/139; 61.1%) were non-synonymous, 43 (43/139;30.9%) were synonymous and one (1/139; 0.7%) was non-sense SNP.

#### Structural variations

Similarly, structural variations (SVs, i.e., insertions or deletions) in H112 and H54 were called relative to reference genome (NC_000962.3). Subsequently, SVs present in H112, but absent in H54 were identified (Table S4). There were 45 SVs present in H112 only. Out of 45, 31 (31/45;68.8%) were present within coding sequences, seven (7/45;15.5%) within repeat regions and another seven (7/45;15.5%) within intergenic regions.

### Comparison of H112 with global circulating strains

#### Phylogeny

**Figure 4:**
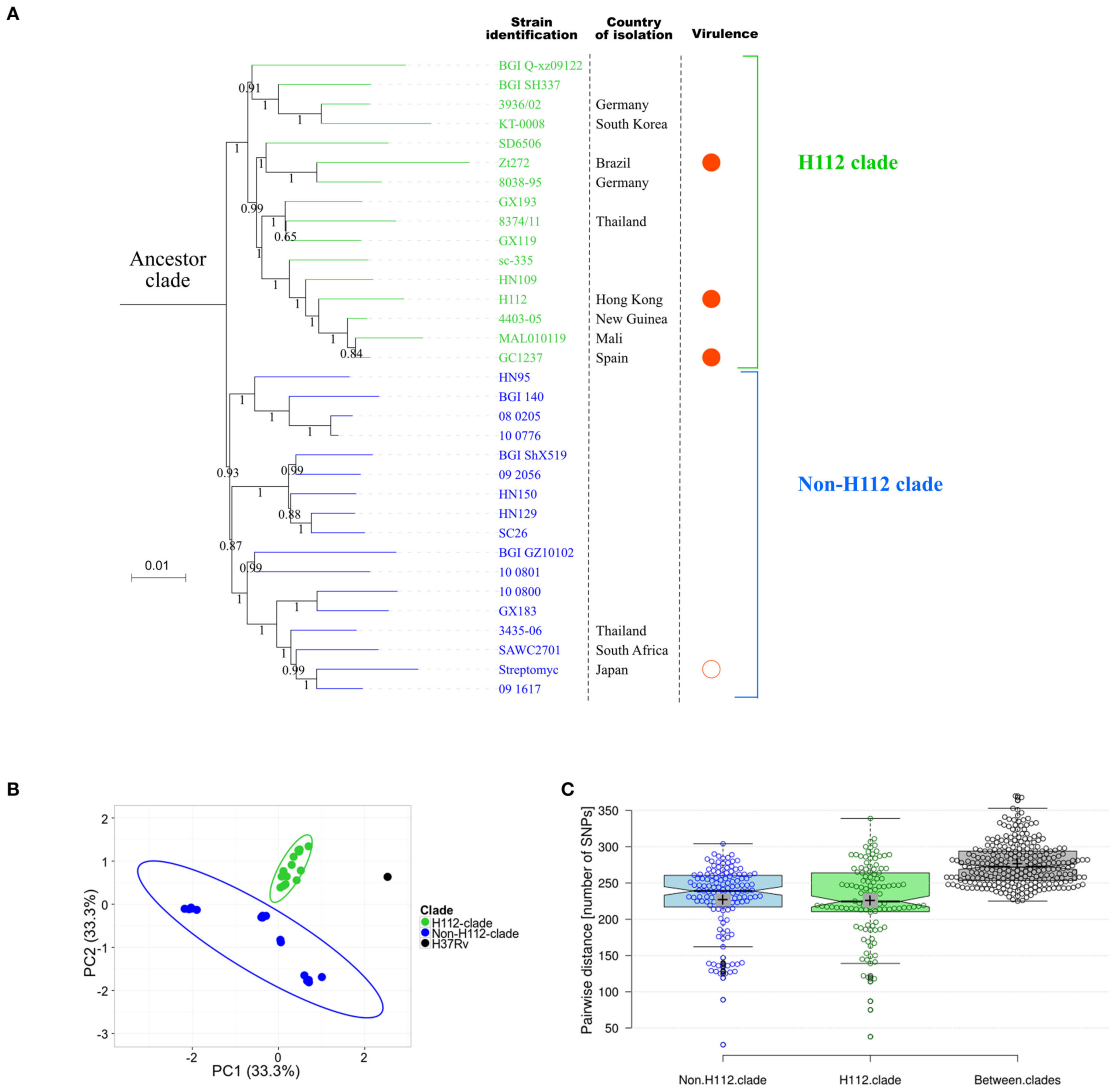
Extensive genomic comparisons between H112 and previously published genome sequences. **(A)** Maximum-likelihood phylogeny based on previously published genomes related to H112. Branch labels indicate bootstrap values on ratio scale 0–1. Scale bar indicates nucleotide substitutions per site. Strain identifications, country of isolation, and virulence is indicated adjacent to the corresponding tips. Country of isolation is mainland China unless otherwise indicated. For virulence column, filled circles represent high virulence and empty circle represent low virulence. No circle is drawn in case of unknown virulence. **(B)** Results of principal component analysis showing clear distinction between identified clades. Each dot represent a sequence used in the analysis. An ellipse is drawn to show distinction between H112 and non-H112 clade. **(C)** A Tukey boxplot showing comparison of pair-wise SNP differences within H112-clade, non-H112-clade and between them. Box covers 25 and 75th percentile of the data. Center line indicates median and cross indicates mean. Each circle represents a pairwise comparison in bell-swarm representation {*n* = 136 (non-H112-clade), 120 (H112-clade), 272 (Between-clades)}.

For a more comprehensive comparison of H112 with global circulating strains, mutations present in H112, but absent in H54, were used to screen public genomes. Our search identified 33 strains collected from 13 countries that also harbored at least one of these mutations. WGS-based phylogeny clustered them into one large clade (ancestor clade) that could be further divided into two distinct clades (H112-clade and non-H112-clade) (Figure 4).

A small number of H112-specific SNPs (9/139; 6.4%), were shared among all members of ancestor clade. These included mutation in mce1 operon (yrbE1b p.A16T) and DNA-repair pathway (ogt p.R37L) (Table S3). No structural variation was common among all strains of ancestor clade (Table S4).

Notably, other than the nine SNPs common among ancestor clade, a group of 16 strains were found to share a total of 12 (12/139; 8.6%) H112-specific SNPs and five (5/45; 11.1%) H112-specific SVs, and were therefore classified as a distinct clade, which was named as H112-clade (Figure 4).

#### Other highly virulent strains related to H112

In addition to hypervirulent strain H112 described in this study, three other highly virulent strains clustered within H112-clade. These were *M. tuberculosis GC1237* resulting in multiple outbreaks in Gran Canaria, Spain (Caminero et al., 2001; Alonso et al., 2011). *M. tuberculosis Zt272* that resulted in increased bacterial load in lungs, decreased survival time and increased area of pneumonia relative to other strains in the intra-tracheally infected mice (Ribeiro et al., 2014). The *M. tuberculosis* Zaragoza strain caused outbreak in Zaragoza, Spain, and reached 18.7% of all isolates of *M. tuberculosis* in 2001 to 2004 (Jia et al., 2017; Rodríguez-Castillo et al., 2017). On the contrary, non-H112 clade strain included the *streptomycin-dependent M. tuberculosis 18b* strain, which has been reported to be severely attenuated in high-dose (>1,000 bacilli) aerosol infected mice model (Campos-Neto, 2016) although the virulence of other strains in this clade have not been phenotypically characterized.

#### Mutations common between H112 and other highly virulent strains

##### Single nucleotide polymorphisms

There were 12 SNPs specific to the H112-clade: one (1/12;8.3%) intergenic, three (3/12;25%) synonymous, and eight (8/12;66.6%) non-synonymous SNPs (Table 2). Only one SNP (1/12;8.3%), Rv0178 (p. D150E), was predicted to have a deleterious impact on the protein function (SIFT score <0.05). Interestingly, Rv0178 is encoded within mammalian cell entry 1 (*mce1)* operon and has been implicated in modulating mycobacterial virulence.

**Table 2:** Single nucleotide polymorphisms common to H112-clade.

**Position (NC_000962.3)**	**Nucleotide change**	**Gene**	**Type**	**Amino acid change**	**SIFT prediction**
209387	T>G	*rv0178*	Non-synonymous	D150E	Deleterious
3865243	G>T	*eccC4*	Non-synonymous	A999D	Tolerated
3012950	G>T	*rv2696c*	Non-synonymous	A220E	Tolerated
249350	G>A	*rv0209*	Non-synonymous	A105T	Tolerated
3785898	G>A	*rv3371*	Non-synonymous	A323T	Tolerated
2201808	C>G	*higB*	Non-synonymous	D30E	Tolerated
3301648	T>G	*fadD29*	Non-synonymous	M270L	Tolerated
1622580	C>A	*rv1443c*	Non-synonymous	R38L	Tolerated
3569220	G>A	*uvrD2*	Synonymous	A664A	Tolerated
752134	C>T	*mkl*	Synonymous	I206I	Tolerated
3476350	G>A	*agpS*	Synonymous	S204S	Tolerated
295746	C> T	*fadE5-rv0245*	Intergenic	–	–

##### Structural variations

Of the five SVs common among members of H112–clade, three (3/5;60%) were located within coding sequences, two (2/5;40%) were present within intergenic regions (Table 3). Among the coding sequences affected by SVs, two (*rv2286c* and *rv0840c*) were disrupted by *IS6110* insertion events. In addition, there was a 9 bp deletion within a possible exported protein *rv0633c*. Both intergenic SVs were small deletions (i.e., less than three base-pairs). One of them was present between *rv0759c* and *rv0760c*, downstream of key virulence-associated operon *phoPR*. While, another one was in close proximity to *IS6110* fragment, between *rv2168c* and *rv2169c*.

**Table 3:** Structural variation common to H112-clade.

**Start (NC_000962.3)**	**End (NC_000962.3)**	**Size (bp)**	**Type**	**Annotation**
730085	730094	9	Deletion	The deletion was within coding sequence *rv0633c*. *rv0633c* encode possible exported protein and belong to cell wall and cell processes functional category.
854259	854261	2	Deletion	Intergenic:*rv0759c*-*rv0760c*
937115	937115	1,358	Insertion	The insertion was within coding sequence proline iminopeptidase (*pip)*. This insertion event was mediated by *IS6110* element. *Pip* belongs to intermediary metabolism and respiration functional category.
2431514	2431515	1	Deletion	Intergenic:*rv2168c*-*rv2169c*
2559504	2559504	1,358	Insertion	The insertion is within *rv2286c* which encodes a conserved hypothetical protein. This insertion was mediated by *IS6110* element.

## Discussion

In this study, comparative whole genomic analysis between a hypervirulent *M. tuberculosis* clinical strain H112 and another clinical strain H54 of lower virulence from the same lineage was conducted to identify a set of mutations that are specific to the hypervirulent strain. Based on these mutations, we analyzed the phylogenetic relatedness of H112 with all available *M. tuberculosis* whole genomes, which led us to identify a novel phylogenetic cluster that encompassed H112 and the highly virulent strains reported elsewhere. All these strains shared a total of 12 SNPs and five SVs, which cannot be found in other strains with low-level virulence in L2 lineage. This is the first study to report the use of these genetic markers for successful clustering of highly virulent *M. tuberculosis* strains isolated from different parts of the world.

While several studies have suggested that modern phylogenetic groups of L2 (L2.2.1.1 and L2.2.1.2) are more virulent than ancient ones (L2.1, L2.2.1, L2.2.2) (Hanekom et al., 2010; Ribeiro et al., 2014), the current study has identified a highly virulent strain cluster within an ancient group (L2.2.1). In particular, our data point-toward existence of a highly virulent clade, H112-clade, comprising of at least three whole-genome sequenced hypervirulent strains, namely H112, GC1237, and Zt272. Another outbreak strain *M. tuberculosis Zaragoza (MTZ)* also harbors genetic markers of H112-clade, notably, *IS6110* insertion within *rv2286c* and *ogt* (p.R36L) (López-Calleja et al., 2007), and thus could also be linked to H112-clade although whole-genome sequence for this strain has yet to be available. The enhanced virulence of these strains was previously demonstrated in individual studies and was shown to be independent of each other. In this study, through an extensive phylogenetic analysis, these strains were shown to be closely related to one another. The close relationship between these highly virulent strains suggested that genetic factors common to them are likely to contribute toward their hypervirulence, although the causative effect of these mutations should be further investigated in the future study.

WGS has emerged as a powerful tool to identify genetic determinants of mycobacterial virulence. Notably, comparative genomics approach has revealed possible associations between genetic polymorphisms and virulence diversity in *M. tuberculosis* (Jia et al., 2017; Rodríguez-Castillo et al., 2017). However, virulence of the *M. tuberculosis* strains used in most of these studies was largely unknown. In addition, no effort was made to eliminate lineage-related mutations, leading to identification of numerous genetic mutations that could be irrelevant to mycobacterial virulence. Conversely, in the present study, hypervirulence of strain H112 was well-defined by enhanced survivability in macrophage models of *M. tuberculosis* infection as well as severe clinical manifestation (TB meningitis) in a 51-year old immunocompetent patient. By genomic comparison with a less-virulent strain from same phylogenetic lineage, the hypervirulent strain-specific mutations were distinguished from the massive lineage-related mutations. These mutations were then used to screen for public genomes, and those which were commonly shared by hypervirulent *M. tuberculosis* strains isolated from different global regions were considered as potential genetic determinants of mycobacterial virulence. This analysis cascade helped us to pinpoint 12 SNPs out of 1,238 SNPs obtained immediately from WGS of H112.

The impact of the interested SNPs on protein function was further predicted by computational algorithm. Only one non-synonymous mutation, D150E, in *rv0178* within mce1 operon was predicted to be deleterious. The mce1 operon forms a cluster of 13 genes encoding two Yrb-like permeases (YrbE1A and YrbE1B), six core mce proteins (Mce1A-F), fatty-acid-CoA ligase (FadD5), and four conserved hypothetical proteins (Rv0175-78) (Shimono, 2003). Deletion of mce1A or yrbE1B has been shown to abolish expression of other mce1 proteins as well (Shimono, 2003). Intravenous infection with mce1A or yrbE1B deleted strain of *M. tuberculosis* (analogous to complete absence of mce1 operon) was shown to result in increased bacillary load and poorly organized granulomas in mice (Shimono, 2003). It is interesting to speculate that deleterious mutation within mce1 operon, identified here, might be one of the contributing factors to increased virulence of H112-clade.

It was also observed that a 2 bp deletion in the intergenic region *rv0759c*-*rv0760c*, was present in all members of H112-clade. Both *rv0759c* and *rv0760c* encode conserved hypothetical proteins with unknown function. The intergenic deletion was located~400 bp downstream of *phoPR* operon. PhoPR is a two-component system that plays an important role in mycobacterial virulence. A deleterious SNP in *phoP* has been demonstrated as one of the reasons for avirulence of strain H37Ra (Lee et al., 2008). On the other hand, a promoter mutation linked with an increased *phoP* expression was found in an outbreak strain of *M. bovis* (Soto et al., 2004). It is well-known that downstream gene variants could also modulate the expression level of upstream gene, particularly, by altering the binding sites for transcriptional regulators. Interestingly, the intergenic region *rv0759c*-*rv0760c* has been identified as a binding target for an alternative sigma factor, Sigma F (Rodrigue et al., 2007), which is a regulator of *phoP* (Hümpel et al., 2010). We hypothesize that in H112-clade, deletion within regulatory region *rv0759c*-*rv0760c*, might confer hypervirulence by modulating expression of upstream gene *phoP*.

The current study was still limited in several aspects. First, the study was focused on only one hypervirulent strain and a control strain. However, both strains were selected from a cohort of more than one hundred strains after extensive genetic and virulence comparisons. The genome-sequence of hypervirulent strain H112 was also compared with other highly virulent strains to overcome limitations associated with sample size. Second, virulence of strains H112 and H54, was not determined in animal models of *M. tuberculosis* infection. However, an alternative model of *M. tuberculosis* infection, human macrophage, which is also well-established in the field, was used to assay virulence. The results were further interpreted in context of clinical background of strains. Third, the information regarding the virulence of *M. tuberculosis* other than the four hypervirulent strains in H112-clade were not available. The accuracy of using the interested mutations as epidemiological markers for tracing hypervirulent *M. tuberculosis* strains therefore required further investigation.

Most importantly, the functional impacts of H112-clade-specific mutations have not been experimentally validated. In the future study, their causative roles in mycobacterial virulence should be determined through genetic manipulation. For instance, the mutations could be introduced into the wildtype *M. tuberculosis* genome using allelic exchange approach (Gopinath et al., 2015), or alternatively, the wildtype genes could be transformed into H112 to compensate the respective mutated gene (Siu et al., 2014). The virulence of the genetically manipulated strains should be assessed in cell culture and mice models of *M. tuberculosis* infection.

In the present study, we identified a novel phylogenetic clade that encompassed highly virulent *M. tuberculosis* strains isolated from different geographic regions. The genetic mutations common to them may explain the mechanism underlying the enhanced virulence of these strains. The causative effect of these mutations on mycobacterial virulence should be experimentally validated in the future study.

## Author contributions

Conceived and designed the experiments: RR, WY, YZ, and GS. Performed the experiments: RR, BW, KL, KKT, KTT, LZ, and GS. Analyzed the data: RR, YZ, YK, and GS. Contributed reagents, materials, analysis tool: RR, WY, KTT, LZ, and GS. Wrote the paper: RR, WY, YZ, YK, KL, and GS.

### Conflict of interest statement

The authors declare that the research was conducted in the absence of any commercial or financial relationships that could be construed as a potential conflict of interest.

## References

[B1] Adams M. D. Bishop B. Wright M. S. (2016). Quantitative assessment of insertion sequence impact on bacterial genome architecture. Microb. Genomics 2:e000062. 10.1099/mgen.0.000062 28348858 PMC5343135

[B2] Alonso H. Aguilo J. I. Samper S. Caminero J. A. Campos-Herrero M. I. Gicquel B.. (2011). Deciphering the role of IS6110 in a highly transmissible *Mycobacterium tuberculosis* Beijing strain, GC1237. Tuberculosis 91, 117–126. 10.1016/j.tube.2010.12.007 21256084

[B3] Benjak A. Sala C. Hartkoorn R. C. (2015). Whole-genome sequencing for comparative genomics and *de novo* genome assembly. Methods Mol. Biol. 1285, 1–16. 10.1007/978-1-4939-2450-9_1 25779307

[B4] Caminero J. A. Pena M. J. Campos-Herrero M. I. Rodriguez J. C. Garcia I. Cabrera P.. (2001). Epidemiological evidence of the spread of a *Mycobacterium tuberculosis* strain of the Beijing genotype on Gran Canaria Island. Am. J. Respir. Crit. Care Med. 164, 1165–1170. 10.1164/ajrccm.164.7.2101031 11673204

[B5] Campos-Neto A. (2016). *Mycobacterium tuberculosis* strain 18b, a useful non-virulent streptomycin dependent mutant to study latent tuberculosis as well as for *in vivo* and *in vitro* testing of anti-tuberculosis drugs. Tuberculosis 99, 54–55. 10.1016/j.tube.2016.04.006 27450005

[B6] Chin C. S. Alexander D. H. Marks P. Klammer A. A. Drake J. Heiner C.. (2013). Nonhybrid, finished microbial genome assemblies from long-read SMRT sequencing data. Nat. Methods 10, 563–569. 10.1038/nmeth.2474 23644548

[B7] Coll F. McNerney R. Guerra-Assuncao J. A. Glynn J. R. Perdigao J. Viveiros M.. (2014). A robust SNP barcode for typing *Mycobacterium tuberculosis* complex strains. Nat. Commun. 5:4812. 10.1038/ncomms5812 25176035 PMC4166679

[B8] Cowan L. S. Mosher L. Diem L. Massey J. P. Crawford J. T. (2002). Variable-number tandem repeat typing of *Mycobacterium tuberculosis* isolates with low copy numbers of IS6110 by using mycobacterial interspersed repetitive units. J. Clin. Microbiol. 40, 1592–1602. 10.1128/JCM.40.5.1592-1602.2002 11980927 PMC130938

[B9] Gopinath K. Warner D. F. Mizrahi V. (2015). Targeted gene knockout and essentiality testing by homologous recombination. Methods Mol. Biol. 1285, 131–149. 10.1007/978-1-4939-2450-9_8 25779314

[B10] Hanekom M. Mata D. van Pittius N. G. van Helden P. Warren R. Hernandez-Pando R. (2010). *Mycobacterium tuberculosis* strains with the Beijing genotype demonstrate variability in virulence associated with transmission. Tuberculosis 90, 319–325. 10.1016/j.tube.2010.08.004 20832364

[B11] Hümpel A. Gebhard S. Cook G. M. Berney M. (2010). The SigF regulon in *Mycobacterium smegmatis* reveals roles in adaptation to stationary phase, heat, and oxidative stress. J. Bacteriol. 192, 2491–2502. 10.1128/JB.00035-10 20233930 PMC2863567

[B12] Jia X. Yang L. Dong M. Chen S. Lv L. Cao D.. (2017). The Bioinformatics analysis of comparative genomics of *Mycobacterium tuberculosis* Complex (MTBC) provides insight into dissimilarities between intraspecific groups differing in host association, virulence, and epitope diversity. Front. Cell. Infect. Microbiol. 7:88. 10.3389/fcimb.2017.00088 28377903 PMC5360109

[B13] Kamerbeek J. Schouls L. Kolk A. Van Agterveld M. Van Soolingen D. Kuijper S.. (1997). Simultaneous detection and strain differentiation of *Mycobacterium tuberculosis* for diagnosis and epidemiology. J. Clin. Microbiol. 35, 907–914. 9157152 10.1128/jcm.35.4.907-914.1997 PMC229700

[B14] Kumar P. Henikoff S. Ng P. C. (2009). Predicting the effects of coding non-synonymous variants on protein function using the SIFT algorithm. Nat. Protoc. 4, 1073–1081. 10.1038/nprot.2009.86 19561590

[B15] Kumar S. Stecher G. Tamura K. (2016). MEGA7: Molecular Evolutionary Genetics Analysis version 7.0 for bigger datasets. Mol. Biol. Evol. 33, 1870–1874. 10.1093/molbev/msw054 27004904 PMC8210823

[B16] Lee J. S. Krause R. Schreiber J. Mollenkopf H. J. Kowall J. Stein R.. (2008). Mutation in the transcriptional regulator PhoP contributes to avirulence of *Mycobacterium tuberculosis* H37Ra strain. Cell Host Microbe 3, 97–103. 10.1016/j.chom.2008.01.002 18312844

[B17] Li H. Handsaker B. Wysoker A. Fennell T. Ruan J. Homer N.. (2009). The sequence alignment/map format and SAMtools. Bioinformatics 25, 2078–2079. 10.1093/bioinformatics/btp352 19505943 PMC2723002

[B18] López-Calleja A. Lezcano M. Vitoria M. Iglesias M. Cebollada A. Lafoz C.. (2007). Genotyping of *Mycobacterium tuberculosis* over two periods: a changing scenario for tuberculosis transmission. Int. J. Tuberculosis Lung Dis. 11, 1080–1086. 17945064

[B19] Luo T. Comas I. Luo D. Lu B. Wu J. Wei L.. (2015). Southern East Asian origin and coexpansion of *Mycobacterium tuberculosis* Beijing family with Han Chinese. Proc. Natl. Acad. Sci. U.S.A. 112, 8136–8141. 10.1073/pnas.1424063112 26080405 PMC4491734

[B20] Merker M. Blin C. Mona S. Duforet-Frebourg N. Lecher S. Willery E.. (2015). Evolutionary history and global spread of the *Mycobacterium tuberculosis* Beijing lineage. Nat. Genet. 47, 242–249. 10.1038/ng.3195 25599400 PMC11044984

[B21] Nattestad M. Schatz M. C. (2016). Assemblytics: a web analytics tool for the detection of variants from an assembly. Bioinformatics 32, 3021–3023. 10.1093/bioinformatics/btw369 27318204 PMC6191160

[B22] Quinlan A. R. Hall I. M. (2010). BEDTools: a flexible suite of utilities for comparing genomic features. Bioinformatics 26, 841–842. 10.1093/bioinformatics/btq033 20110278 PMC2832824

[B23] Ribeiro S. C. Gomes L. L. Amaral E. P. Andrade M. R. Almeida F. M. Rezende A. L.. (2014). *Mycobacterium tuberculosis* strains of the modern sublineage of the Beijing family are more likely to display increased virulence than strains of the ancient sublineage. J. Clin. Microbiol. 52, 2615–2624. 10.1128/JCM.00498-14 24829250 PMC4097719

[B24] Rodrigue S. Brodeur J. Jacques P.-É. Gervais A. L. Brzezinski R. Gaudreau L. (2007). Identification of mycobacterial σ factor binding sites by chromatin immunoprecipitation assays. J. Bacteriol. 189, 1505–1513. 10.1128/JB.01371-06 17158685 PMC1855719

[B25] Rodríguez-Castillo J. G. Pino C. Niño L. F. Rozo J. C. Llerena-Polo C. Parra-López C. A.. (2017). Comparative genomic analysis of *Mycobacterium tuberculosis* Beijing-like strains revealed specific genetic variations associated with virulence and drug resistance. Infect. Genet. Evol. 54, 314–323. 10.1016/j.meegid.2017.07.022 28734764

[B26] Shabbeer A. Cowan L. S. Ozcaglar C. Rastogi N. Vandenberg S. L. Yener B.. (2012). TB-Lineage: an online tool for classification and analysis of strains of *Mycobacterium tuberculosis* complex. Infect. Genet. Evol. 12, 789–797. 10.1016/j.meegid.2012.02.010 22406225

[B27] Shimono N. (2003). Hypervirulent mutant of *Mycobacterium tuberculosis* resulting from disruption of the mce1 operon. Proc. Natl. Acad. Sci. U.S.A. 100, 15918–15923. 10.1073/pnas.2433882100 14663145 PMC307668

[B28] Siu G. K. H. Yam W. C. Zhang Y. Kao R. Y. (2014). An upstream truncation of the furA-katG operon confers high-level isoniazid resistance in a *Mycobacterium tuberculosis* clinical isolate with no known resistance-associated mutations. Antimicrob. Agents Chemother. 58, 6093–6100. 10.1128/AAC.03277-14 25092698 PMC4187958

[B29] Siu G. K. Tam Y. H. Ho P. L. Lee A. S. Que T. L. Tse C. W.. (2011). Direct detection of isoniazid-resistant *Mycobacterium tuberculosis* in respiratory specimens by multiplex allele-specific polymerase chain reaction. Diagn. Microbiol. Infect. Dis. 69, 51–58. 10.1016/j.diagmicrobio.2010.08.021 21146714

[B30] Smith I. (2003). *Mycobacterium tuberculosis* pathogenesis and molecular determinants of virulence. Clin. Microbiol. Rev. 16, 463–496. 10.1128/CMR.16.3.463-496.2003 12857778 PMC164219

[B31] Soto C. Y. Menéndez M. C. Pérez E. Samper S. Gómez A. B. García M. J.. (2004). IS6110 mediates increased transcription of the phoP virulence gene in a multidrug-resistant clinical isolate responsible for tuberculosis outbreaks. J. Clin. Microbiol. 42, 212–219. 10.1128/JCM.42.1.212-219.2004 14715755 PMC321672

[B32] Treangen T. J. Ondov B. D. Koren S. Phillippy A. M. (2014). The Harvest suite for rapid core-genome alignment and visualization of thousands of intraspecific microbial genomes. Genome Biol. 15:524. 10.1186/s13059-014-0524-x 25410596 PMC4262987

[B33] Weniger T. Krawczyk J. Supply P. Niemann S. Harmsen D. (2010). MIRU-VNTRplus: a web tool for polyphasic genotyping of *Mycobacterium tuberculosis* complex bacteria. Nucleic Acids Res. 38(Web Server issue), W326–W331. 10.1093/nar/gkq351 20457747 PMC2896200

[B34] Wong K. C. Leong W. M. Law H. K. Ip K. F. Lam J. T. Yuen K. Y.. (2007). Molecular characterization of clinical isolates of *Mycobacterium tuberculosis* and their association with phenotypic virulence in human macrophages. Clin. Vaccine Immunol. 14, 1279–1284. 10.1128/CVI.00190-07 17715326 PMC2168117

[B35] Zhang H. Li D. Zhao L. Fleming J. Lin N. Wang T.. (2013). Genome sequencing of 161 *Mycobacterium tuberculosis* isolates from China identifies genes and intergenic regions associated with drug resistance. Nat. Genet. 45, 1255–1260. 10.1038/ng.2735 23995137

