# Supplementary material for: Comparative Whole-Genomic Analysis of an Ancient L2 Lineage Mycobacterium tuberculosis Reveals a Novel Phylogenetic Clade and Common Genetic Determinants of Hypervirulent Strains
Source: Front Cell Infect Microbiol. 2018 Jan 12;7:539. doi: 10.3389/fcimb.2017.00539 (PMC5770396; doi:10.3389/fcimb.2017.00539)
Supplement: Supplementary file 5 [file Image1.PDF]

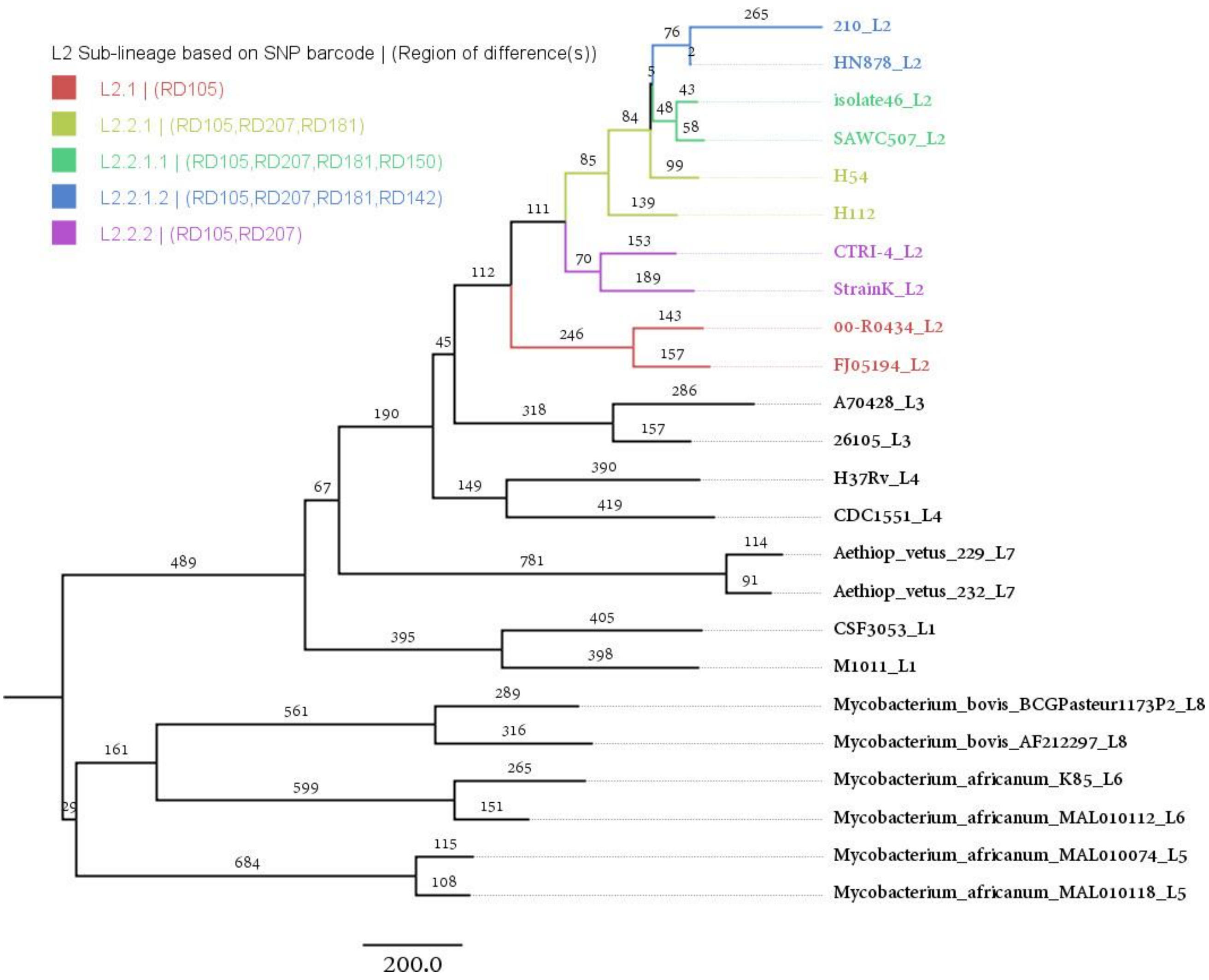

Supplementary Figure 1. Assignment of phylogenetic lineage to H112 and H54 is illustrated using neighbour-joining (NJ) phylogeny with representative strains from all eight major lineages (L1 – L8) of M. tuberculosis complex (MTBC) and L2 sub-lineages. Scale bar and branch labels indicate number of SNPs.
